# Combinatorial and Machine Learning Approaches for Improved Somatic Variant Calling From Formalin-Fixed Paraffin-Embedded Genome Sequence Data

**DOI:** 10.3389/fgene.2022.834764

**Published:** 2022-04-27

**Authors:** Dollina D. Dodani, Matthew H. Nguyen, Ryan D. Morin, Marco A. Marra, Richard D. Corbett

**Affiliations:** ^1^ The Bioinformatics Graduate Program, University of British Columbia, Vancouver, BC, Canada; ^2^ Canada’s Michael Smith Genome Sciences Centre, BC Cancer Research Institute, Provincial Health Services Authority, Vancouver, BC, Canada; ^3^ Department of Molecular Biology and Biochemistry, Simon Fraser University, Burnaby, BC, Canada; ^4^ Department of Medical Genetics, University of British Columbia, Vancouver, BC, Canada

**Keywords:** FFPE (formalin fixed paraffin-embedded), whole genome, somatic variant calling, combinatorics, machine learning

## Abstract

Formalin fixation of paraffin-embedded tissue samples is a well-established method for preserving tissue and is routinely used in clinical settings. Although formalin-fixed, paraffin-embedded (FFPE) tissues are deemed crucial for research and clinical applications, the fixation process results in molecular damage to nucleic acids, thus confounding their use in genome sequence analysis. Methods to improve genomic data quality from FFPE tissues have emerged, but there remains significant room for improvement. Here, we use whole-genome sequencing (WGS) data from matched Fresh Frozen (FF) and FFPE tissue samples to optimize a sensitive and precise FFPE single nucleotide variant (SNV) calling approach. We present methods to reduce the prevalence of false-positive SNVs by applying combinatorial techniques to five publicly available variant callers. We also introduce FFPolish, a novel variant classification method that efficiently classifies FFPE-specific false-positive variants. Our combinatorial and statistical techniques improve precision and F1 scores compared to the results of publicly available tools when tested individually.

## Introduction

While formalin-fixed, paraffin-embedded (FFPE) tissues are routinely used for clinical purposes, many next-generation sequencing studies rely on whole blood or fresh frozen (FF) tissues to yield the best results (Haile et al., 2019). Due to difficulties associated with fresh tissue procurement, the infrastructure required for its retention, and the suitability of FFPE tissues for routine pathology assays, FFPE remains the preferred method for storing clinical samples (Robbe et al., 2018a). Unfortunately, the fixation process used to produce FFPE samples creates nucleic acid damage that presents unique challenges to accurate and comprehensive whole-genome sequencing (WGS) analyses (Robbe et al., 2018a; Haile et al., 2019). Methods to improve the extraction of nucleic acids from FFPE tissues have emerged (Haile et al., 2017; Haile et al., 2019). Still, FFPE-induced artifacts, such as formaldehyde crosslinks, DNA fragmentation, abasic sites, and deamination of cytosine bases (Do and Dobrovic, 2015; Haile et al., 2017), remain problematic and can confound the identification of somatic single nucleotide variants (SNVs). Approaches to filter or otherwise lessen the effects of these artifacts while retaining true sequence variants are thus needed. Several studies have benchmarked existing SNV callers on data from FFPE samples (Robbe et al., 2018b; Xu, 2018), and a recent tool is available to filter split-reads (Wei et al., 2021). Still, relatively little work has been done to ameliorate artifacts in genome sequence data (de Schaetzen van Brienen et al., 2020).

In this study, we analyzed WGS data from FFPE and matched FF tissues to identify methods that yield high-quality somatic FFPE-derived SNVs. We first tested available somatic callers individually and subsequently in combination to evaluate the extent to which recall, precision, and F1 score could be improved. We describe FFPolish, a new tool that, to the best of our knowledge, is the first open-source machine learning-based method for filtering FFPE somatic SNV calls.

## Methods

Lacking orthogonal FFPE ground truth against which to compare SNV results obtained using different computational approaches, we chose instead to use SNV data from FF material prepared from the same patient tumours. WGS data from peripheral normal blood samples, along with matched FFPE and FF tumour WGS data, were used to identify somatic SNVs. We compared somatic SNVs called from the FFPE material to somatic SNVs from the matched FF material for each patient. This approach allowed us to identify variants either unique to the FFPE samples (candidate FFPE false positives) or unique to the FF tissue (candidate FFPE false negatives). We used cancer patient samples from Human Tumour Molecular Characterization Project (HTMCP) (Gagliardi et al., 2020) and Burkitt Lymphoma Genome Sequencing Project (BLGSP) (Grande et al., 2019) for this study (Table 1).

**TABLE 1:** Summary of the BLGSP and HTMCP samples. Fold redundancy of genome sequencing coverage (X) is indicated*.*

	HTMCP	BLGSP
Genome Reference	hg19	hg38
FFPE Tumour	N = 39; HiSeq 2500; 50.3X	N = 34; HiSeq X; 68.9X
FF Tumour	N = 39; HiSeq 2500; 82.5X	N = 34; HiSeq 2500; 82.4X
FF Normal	N = 39; HiSeq 2500; 42.8X	N = 34; HiSeq 2500; 41.0X

### Samples and Extraction

Cervical cancer and Burkitt lymphoma cases were selected from the HTMCP and the BLGSP projects. The HTMCP cervical cancer samples were obtained from female patients treated at the Uganda Cancer Institute in Kampala, Uganda. The BLGSP samples were obtained from Uganda and the United States of America. Both the HTMCP cervical and BLGSP tumour samples underwent rigorous pathology consensus review.

The Fred Hutchinson Cancer Research Center Institutional Review Board (protocols #U009 and #7662), in consultation with the Uganda Cancer Institute and the government of Uganda, approved the accrual of both BLGSP and HTMCP samples. Informed consent was obtained from all participants. The molecular characterization protocol was approved by the BC Cancer Research Ethics Board (UBC BC Cancer REB - certificate number H16-02279).

### Whole-Genome Sequencing, Analysis, and Alignment of FF and FFPE Samples

Whole-genome sequencing library construction for BLGSP (FF and FFPE) samples was performed on DNA provided by Nationwide Children’s Hospital (Columbus, OH). Nucleic acids from HTMCP tissue samples were extracted at Canada’s Michael Smith Genome Sciences Centre, BC Cancer (Vancouver, BC). For the BLGSP FFPE samples, we constructed sequencing libraries from FFPE-derived DNA as described (Grande et al., 2019). Briefly, solid-phase reversible immobilization (SPRI) bead-based size selection was performed before library construction to remove smaller DNA fragments from degraded FFPE DNA. HTMCP FFPE sequence libraries were prepared as described in Section 1 of the Supplementary Material. Briefly, 100 ng of FFPE arrayed in each well of a 96-well plate were sheared and subjected to magnetic bead-based size selection. After 3′ A-tailing, libraries were bead-purified and amplified using eight cycles of PCR and primers containing a hexamer index, which enabled library pooling. For the HTMCP and BLGSP FF samples, we implemented a version of the TruSeq DNA PCR-free kit, automated on a liquid handling device as described previously (Grande et al., 2019; Gagliardi et al., 2020).

Fastq files were generated using Bcl2fastq2 2.17.1.14 with default parameters. Alignments were performed after the phasing base was removed from the raw fastq files. BLGSP and HTMCP reads were aligned to hg38 and hg19 human genome references, respectively, using Minimap2 (2.15) (Li, 2018) with parameters "-ar sx”. Duplicates in the BAM files were marked using Sambamba (​0.6.1) (Tarasov et al., 2015).

Metrics including error rate, coverage, insert size, mapping quality, percentage of reads with insertions or deletions and GC bias were estimated and extracted using Qualimap (2.2.1) (García-Alcalde et al., 2012) and Picard (2.4.1)^1^. IGV (Robinson et al., 2011) was used for manual read and alignment inspection.

### Detection of Single Nucleotide Variants, Ground Truth, and Benchmarking

#### Preliminary Assessment of Variant Callers Using Sample BLGSP-71-06-00001-01B-01E

Each patient sample in our study had an FFPE tumour, FF tumour, and FF normal genome available for analysis. For our feasibility testing, we attempted to call somatic FFPE tumour and FF tumour variants in a single sample using 10 variant callers: LoFreq (Wilm et al., 2012), Pisces (Dunn et al., 2019), SomVarIUS (Smith et al., 2016), Platypus (Rimmer et al., 2014), Shimmer (Hansen et al., 2013), outLyzer (Muller et al., 2016), Strelka2 (Saunders et al., 2012), Virmid (Kim et al., 2013), Octopus (Cooke et al., 2018) and Mutect2 (McKenna et al., 2010). The commands used are in Section 2 of the Supplementary Material. This test measured each tool’s resource requirement and suitability in our study.

After initial testing, we eliminated Pisces, outLyzer, and Octopus from further analysis due to computational requirements beyond what we allocated to this project (Supplementary Table S1). We further eliminated SomVarIUS and Platypus from subsequent testing due to an observed inability to filter germline variants (Section 3 of the Supplementary Material).

#### Complete Cohort Analysis of Five Selected Variant Callers

**FIGURE 1:**
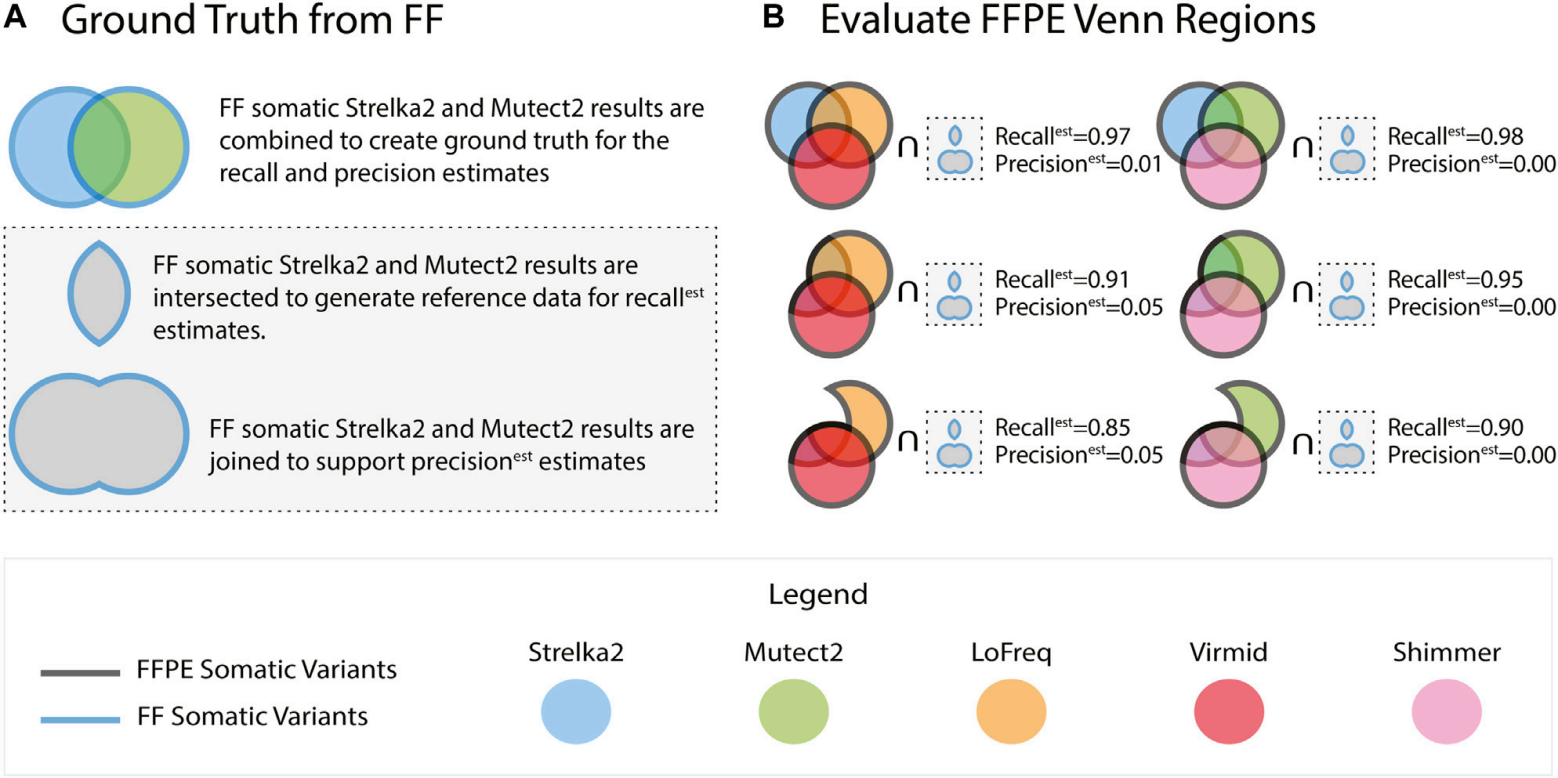
FFPE somatic variants were identified using five callers. Ground truth used to evaluate the FFPE variants is generated using the Strelka2 and Mutect2 variants from FF tumours **(A)**. Recall^est^ and precision^est^ are calculated by comparing against the intersection and the union of the Strelka2 and Mutect2 FF results, respectively. The FFPE results from the five callers are collated into groups of three and intersected in a Venn-like fashion. Each of the 127 possible combinations of the Venn intersection parts is compared against the ground truth **(B)**. The results reported in **(B)** are from sample BLGSP-71-06-00001-01B-01E.

For evaluating each of the 5 callers passing our initial tests, we considered using the FF somatic variant calls from the tool as the ground truth. Still, we reasoned that misleadingly high concordance between the FFPE and FF variants was possible. We note that high concordance could arise due to a preponderance of false positives in the FF results. To address this possibility, we assessed each tool using a compendium of ground truth data assembled from the FF outputs of multiple tools. Specifically, we used the vcf-merge function from rtg-tools (Cleary et al., 2015) to take the union and intersection of the FF somatic variants called by Mutect2 and Strelka2 (Figure 1A) - two tools that have repeatedly been reported to generate high quality somatic SNVs from FF sources (Chen et al., 2020).

$$recall^{est\;}=\;\frac{Tool\;tested\cap \;\left(Mutect2\cap \;Strelka2\right)}{Mutect2\cap \;Strelka2}\;$$ (eq.1)

$$precision^{est}=\frac{Tool\;tested\cap \left(Mutect2\cup Strelka2\right)}{Tool\;tested}$$ (eq.2)

$$F1^{est\;}=Harmonic\;mean\left(recall^{est},\;precision^{est}\right)$$ (eq.3)

The comparison of results from the matched FF and FFPE tissues is not without uncertainty, as the cells in each partition may express non-identical biological signals. This uncertainty motivated a rigorous selection of our performance metrics. Using the intersection of FF variant sets from Strelka2 and Mutect2 (ground truth callers) as the control for precision and recall estimates is the standard approach. This would lead to misclassification of FFPE-derived variants if they were called by one of the two ground truth callers. In this case, a variant called in the FFPE and any one ground truth caller would be classified as a false positive while being present in two of three datasets being compared. To account for this scenario, we introduce recall^est^, precision^est^, and F1^est^ as defined in Equations 1–3, where Mutect2 and Strelka2 are FF variant sets and ∪ , ∩ represent an intersection and union of the two sets involved.

### Combination of Somatic Variant Caller Results and FFPolish

For each patient, variant calls from five tools (LoFreq, Mutect2, Strelka2, Virmid, Shimmer) were each evaluated in isolation and then combined to test for improved FFPE variant calling recall^est,^ precision^est^, and F1^est^. To make the test computationally feasible, ten data sets, each containing results from 3 tools, were used for the analysis. The VCF files from three tools in each combination were intersected using Starfish^2^, a VCF intersection tool that uses rtg-tools, to partition the called SNVs into the parts of a three-way Venn diagram (Figure 1B). One hundred twenty-seven merged VCF files were generated by the vcf-merge function in rtg-tools representing all possible combinations of the seven areas for each three tool three-way Venn diagram (∑^7^_k=1__n_C_k_ = 2^n^ - 1 = 127).

FFPolish is an FFPE variant filtering approach based on a logistic regression model from scikit-learn (Pedregosa, 2011). The model was trained on somatic Strelka2 FFPE calls from both HTMCP cervical and BLGSP cohorts, with a total of 8,698,388 training SNVs. As above, variants were evaluated by comparing FFPE variant calls and the ground truth variants (FF variants from Strelka2 and Mutect2). We trained FFPolish with the most sensitive (Strelka2) and precise (Lofreq) variant caller (See Results and Section 6 of the Supplementary Material); however, we saw a decrease in the median F1^est^ as well as a decrease in flexibility in the LoFreq model compared to using Strelka2 calls (Supplementary Tables S2, S3).

FFPolish includes an optimized hyper-parameterized model generated from 10-fold cross-validation, allowing users to re-train the model as required. Re-training with additional new, labelled data can result in increased performance, as the influence of any batch effects could be minimized. Feature extraction is performed using a modified version of DeepSVR (Ainscough et al., 2018) and bam-readcounts^3^ that require a tumour BAM and a VCF file (either tumour-only or tumour-normal paired) as input. A total of 31 features (Supplementary Table S4) are extracted from the tumour bam are used to classify variants in FFPolish and can be divided into three categories:

• Summary metrics (e.g., tumour variant allele fraction (VAF), tumour depth)
• Read-count metrics (e.g., the number of reads on the negative strand) for both the variant and reference allele
• Read-averaging metrics (e.g., the average base quality of reads) for both the variant and reference allele

**FIGURE 2:**
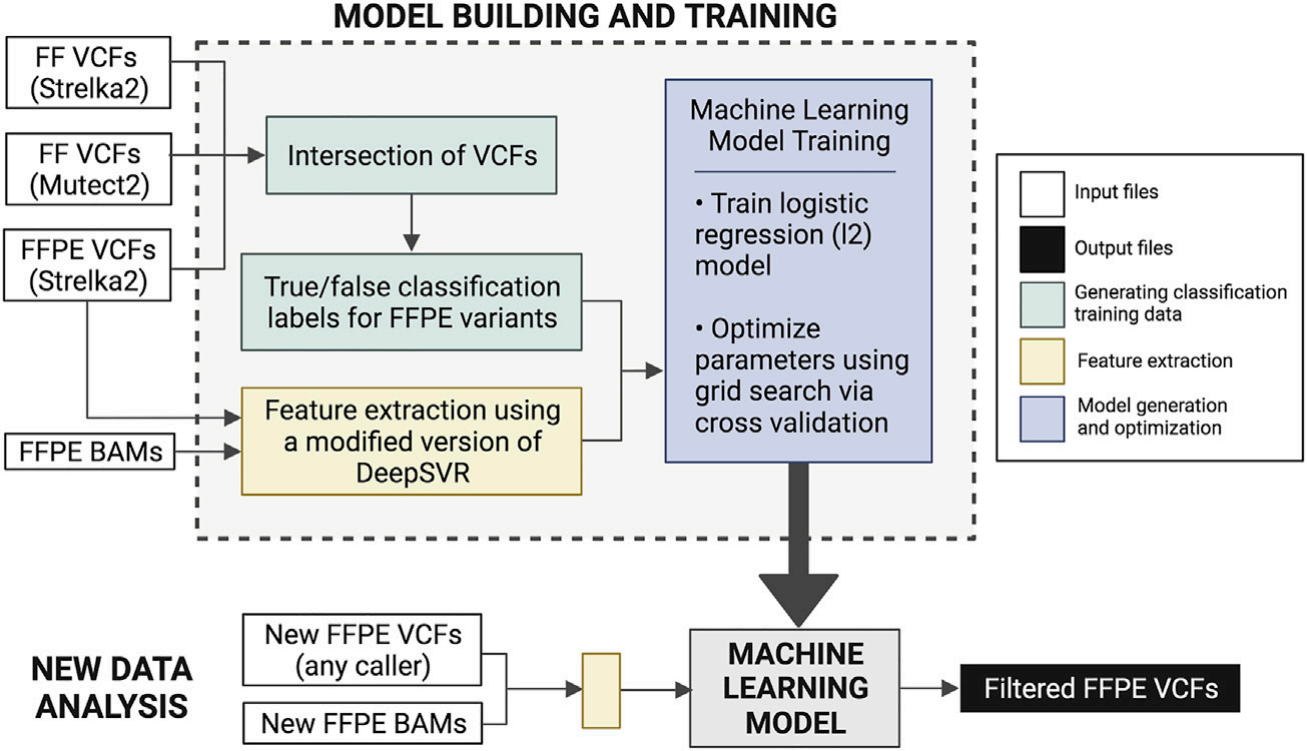
Description of the FFPolish workflow. Generation of the training data was done using Strelka2 FFPE VCFs and the intersection of Strelka2 and Mutect2 FF VCFs. Users may use any somatic variant callers of choice in place of those listed in parentheses. Model training is done using features extracted from FFPE BAM files and Strelka2 FFPE VCF files. The model is built using hyperparameter optimization of logistic regression using grid search and 10-fold cross-validation. The generated model can be applied to any new FFPE VCF and bam file to obtain a filtered FFPE VCF. Users can train a new model if more FFPE data with matched FF results become available in the future.

Logistic regression coefficients were used to assess the importance of the extracted features (Section 4 of the Supplementary Material). Performance validation was conducted through leave-one-out cross-validation to obtain median precision^est^, recall^est^, and F1^est^. Validation was carried out using additional samples (Supplementary Table S5) that were not part of the training data, thus demonstrating generalizability. We applied FFPolish to the previously unseen POG dataset and evaluated the precision^est^, recall^est^, and F1^est^. This validation was also used to compare the generalizability between FFPolish trained with Strelka2 against FFPolish trained with LoFreq (Supplementary Table S6). The FFPolish workflow is described in Figure 2.

## Results

This study aimed to 1) evaluate and improve FFPE SNVcalls through the combination of multiple tools and 2) present an alternative machine learning-based filtering technique fine-tuned to eliminate FFPE artifacts from a single tool.

### Benchmarking Existing SNV Callers on FFPE Samples

We analyzed the overlap between somatic variants using the FFPE and FF tumours to evaluate and optimize the recall^est^ and precision^est^ of somatic SNV calls from FFPE tissue samples. As expected, the number of variants called was tool-dependent (Supplementary Table S7). All FFPE and FF tumours were evaluated for GC bias, background base-error rate, mapping quality, duplicate reads, and the number of mapped reads with insertions or deletion. These metrics were then correlated with each SNV calling performance from each variant caller (Section 5 of the Supplementary Material). In BLGSP, all tools (except Strelka2 and Virmid for recall^est^) showed significant correlations between recall^est^, precison^est^, and the quality metrics. The most significant correlations for precision^est^ and recall^est^ being the qualimap derived percentage of reads with insertions with median Pearson’s correlation of −0.78 (*p-value* 5.00E-08) and −0.410 (*p-value* 1.60E-02), respectively. These correlations were not present in the HTMCP data. Although HTMCP (hg19) and BLGSP (hg38) were extracted at different locations, there were minor differences in the recall^est^ and precision^est^ observed after selected samples were aligned to another reference (Supplementary Table S8).

Tools were tested on FFPE tissue samples using the FF matched normal and then compared to variants in the ground truth data, corresponding to variants called on (FF variants called by tissue data by both Mutect2 and Strelka2). LoFreq and Strelka2 had the highest precision^est^ and recall^est^ outperforming other tools (Section 6 of the Supplementary Material). This indicates that the choice of variant caller can be made based on the metric (recall^est^, precision^est^, or F1^est^) that is most relevant to the user.

### A Combination of Variant Callers Improves Precision^est^ and F1^est^

**FIGURE 3:**
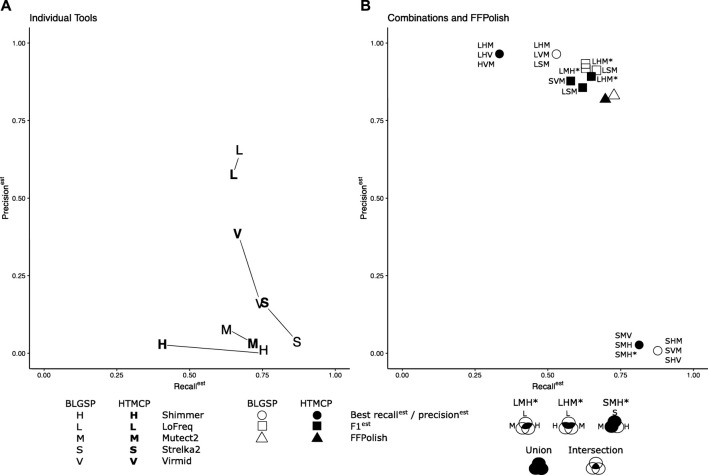
Recall^est^, precision^est^, and F1^est^ of tools tested individually **(A)** compared to the combinations and intersections of tools and FFPolsih **(B)** generated the top three results. Where data points overlap, they have been merged and represented by a single point. For the combinatorial method, a union of the three tools was used for the highest recall^est^ and an intersection for maximum precision^est^ and F1^est,^ as shown in the legend. Special Venn cases have been indicated by * and described in the legend. The regions of the Venn diagram used are shaded in black. The intersection of LoFreq, Shimmer, and Mutect2 resulted in the best precision^est^ for BLGSP (96.89%) and HTMCP 97.78% cohorts. The intersected trios of (LoFreq, Strelka2, Mutect2) and (LoFreq, Shimmer, Mutect2) also obtained the best F1^est^ of 0.770 and 0.751 for BLGSP and HTMCP, respectively. The union of Strelka2, Shimmer and Mutect2 generated the most optimal recall^est^ (87.76%) for BLGSP while the union of Strelka2, Virmid Mutect2 returned the highest recall^est^(81.39%) for HTMCP.

To improve precision^est^ and F1^est^ for somatic SNVs called in FFPE tissues, SNVs identified using multiple callers were combinatorically intersected. We sought to determine the combination and required Venn region intersection of three tools that generated the highest scores (see *Methods Combination of Somatic Variant Caller Results* and Figure 2). We found that different combinations of tools performed best for different sample types (Figure 3).

When tested in isolation for BLGSP samples, Lofreq had the highest precision^est^ and F1^est^ of all individual tools when evaluated across all samples with median values of 65.59 and 0.663, respectively. However, when intersected with Shimmer and Mutect2, median precision^est^ increased to 96.89%. LoFreq intersected with Strelka2, and Mutect2 resulted in the median F1^est^ of 0.770, respectively. Strelka2, in combination with Shimmer and Mutect2, resulted in a median recall^est^ of 87.76% compared to the isolated Strelka2 results (86.86%).

For HTMCP samples, LoFreq intersected with Shimmer and Mutect2, improved the median precision^est^ and F1^est^ across the samples to 97.78% and 0.751, respectively, compared to LoFreq tested individually (57.67%, 0.612). Strelka2, in combination with Virmid and Mutect2, increased median recall^est^ to 81.39%. Based on the choice of combined callers, recall^est^ and precision^est^ were improved for every sample in both sample cohorts, demonstrating that specific combinations could be used to increase the confidence of FFPE variant calls. The top three combinations for recall^est^, precision^est^ and F1^est^ are listed in Supplementary Table S9. Results for all tested combinations are listed in Supplementary Tables S11–S16.

In a recent study, Brienen et al. (2020) used the “at least two” variant calling strategy on nine WGS FFPE samples to improve their SNV calling results. They evaluated four (Strelka2, Mutect2, VarScan2, and Shimmer) variant callers (de Schaetzen van Brienen et al., 2020). Aiming to increase F1 scores, this strategy qualified a variant as putatively positive if called by at least two of the four variant callers. We tested this strategy using the ten combinations of tools on our dataset. These results are summarized in the smaller pie plots in Section 7 of the Supplementary Material. Consistent with the results reported in the Brienen et al. (2020) study, the intersection of the results produced by the combination of Strelka2, Mutect2, and Shimmer resulted in a median F1^est^ of 0.210 (recall^est^ of 0.815, precision^est^ of 0.120) for BLGSP and 0.506 (recall^est^ of 0.725, precision^est^ of 0.389) for HTMCP. These values represent a reduction of 56.0% and 24.6% for BLGSP and HTMCP samples, respectively, from our suggested combinations of LoFreq, Strelka2, and Mutect2 (for BLGSP) and LoFreq, Shimmer, and Mutect2 (for HTMCP).

### FFPolish Achieves Results Comparable to Variant Caller Combinations

To provide an alternative solution to the manual combination of variant callers, a resource and time-intensive task, we developed a machine learning-based approach, namely FFPolish, to refine variants called by any single tool. As shown in Figure 3, FFPolish is a viable alternative to variant caller combinations. It significantly reduces the runtime needed from dozens or hundreds of hours to around the time taken for a single variant caller (Section 8 of the Supplementary Material). FFPolish filters a median of 99.9% of variants from Strelka2 calls, with a median VAF of 0.129 for filtered variants (Supplementary Table S10). For LoFreq calls, FFPolish filters a median of 32.1% of variants, with a median VAF of 0.146 for filtered variants.

By examining the coefficients of FFPolish’s logistic regression model, the most important features can also be determined. Section 4 of the Supplementary Material shows the importance of all 31 features. Overall, some important features are related to reads containing the variant allele and the VAF. The most important feature by a large margin is the average sum of base qualities of mismatches for variant reads. A large negative coefficient can be interpreted such that mismatches (in this case, mismatches are considered as any allele other than the variant allele) of higher base qualities indicate that a called variant may be an artifact.

For BLGSP samples, filtering Strelka2 variant calls using FFPolish obtained a median precision^est^ of 83.08% and a median F1^est^ of 0.775 across all samples when compared to the FF ground truth data (Supplementary Table S3). Compared to the original Strelka2 variant calls, these values represented improvements, with a precision^est^ of 3.71% and F1^est^ of 0.071. Applied to LoFreq calls, FFPolish obtained a median precision^est^ of 95.66% and a median F1^est^ of 0.757, which are an improvement from 65.59% and 0.663 respectively for the original LoFreq variant calls (Supplementary Table S3). FFPolish also performed similarly to the (LoFreq, Shimmer, Mutect2) and (LoFreq, Strelka2, Mutect2) intersections, which produced the best median precision^est^ and F1^est^.

Likewise, for HTMCP samples, FFPolish obtained a median precision^est^ of 81.85% and a median F1^est^ of 0.753, compared to the 16.39% precision^est^ and 0.270 F1^est^ of the original Strelka2 variant calls (Supplementary Table S3). When applied to LoFreq calls, FFPolish obtained a median precision^est^ of 88.86% and a median F1^est^ of 0.727, which are an improvement from 57.67% and 0.612 respectively for the original LoFreq variant calls (Supplementary Table S3). Furthermore, FFPolish performed similarly to the LoFreq, Shimmer, and Mutect2 intersection on the HTMCP data.

FFPolish yielded an overall increase in precision^est^ and F1^est^ for both cohorts compared to unfiltered Strelka2 variant calls. FFPolish is optimized for F1^est^ and therefore did not achieve recall^est^ or precision^est^ scores as high as software combinations and intersections optimized for those metrics. However, the short runtime of FFPolish may be helpful in cases where runtime and computational resources are limiting factors. Running the highest accuracy combinations for BLGSP (LoFreq, Strelka2, and Mutect2) and HTMCP (LoFreq, Shimmer, and Mutect2) required a median of 4936 and 4040 CPU-hours, respectively. In contrast, FFPolish requires only a median of 152 and 135 CPU-hours (including generation of Strelka2 output).

## Discussion

As FFPE samples remain central to clinical diagnostics, methods for confidently calling variants in genomic data derived from such samples are required for enhanced utility in clinical and translational research settings. Our study aims to improve the accuracy of these calls by filtering FFPE caused false-positive variants while retaining the real variants. Separating true variants from sequencing errors is particularly challenging in samples with low tumour content as variant callers may classify variants with low allelic fractions as sequencing errors. Since the allelic fraction of somatic and germline variants will vary with tumour content, standardization of variant calling procedures in diverse FFPE samples represents a challenge (Halperin et al., 2019).

Our findings are consistent with previous studies (Xu, 2018; Halperin et al., 2019) showing that Strelka2 and LoFreq are highly sensitive and precise, respectively. In the HTMCP samples, Mutect2 had a median recall^est^ of 72.04%, which was only ∼3% lower than Strelka2. Notably, Mutect2 did not perform as well with the BLGSP samples, with a median recall^est^ of 63.28%. For studies using a single, well-established somatic variant caller, our findings indicate that LoFreq is best for acquiring high precision^est^ and F1^est^ scores while Strelka2 is best for maximum recall^est^.

To improve confidence in variant calls over what was available from a single caller, we intersected variants from combinations of three tools to maximize estimated recall^est^, precision^est^, and F1^est^. The intersection of LoFreq, Shimmer, and Mutect resulted in the best precision^est^ for BLGSP (96.89%) and HTMCP (97.78%) cohorts. The above intersection combined with calls from [LoFreq ∩ Shimmer] and [LoFreq ∩ Mutect2] for HTMCP obtained the top F1^est^ 0.751. The intersection of LoFreq, Strelka2, and Mutect2 for BLGSP obtained the best F1^est^ 0.770. The union of Strelka2, Shimmer and Mutect2 generated the most optimal recall^est^ (87.76%) for BLGSP while the union of Strelka2, Virmid, Mutect2 returned the highest recall^est^ (81.39%) for HTMCP.

We separately introduce FFPolish, a powerful, machine learning-based post-processing tool that is fine-tuned to eliminate artifactual variant calls from FFPE samples. FFPolish utilizes features such as the read depth, read mapping quality, and read clipped length from FFPE samples, which it obtains directly from VCF and BAM files. The F1 scores obtained by FFPolish for BLGSP (0.775) and HTMCP (0.753) were comparable to the results from combinatorial approaches.

Our comprehensive, rigorous comparison of variant caller performance may allow clinicians and researchers to further rely on whole-genome sequencing data derived from FFPE sources. We have presented options above for the single combinations and intersections of multiple tools and a novel classification method to provide improved recall^est^, precision^est^, or F1^est^ scores.

## Data Availability

The datasets presented in this study can be found in online repositories. The names of the repository/repositories and accession number(s) can be found below: https://portal.gdc.cancer.gov/, phs000527, phs000528, https://ega-archive.org/, EGAZ00001233252, EGAZ00001233267, EGAZ00001233281, EGAZ00001708828, EGAZ00001708850 EGAZ00001233208, EGAZ00001233239, EGAZ00001233274, EGAZ00001708827, EGAZ00001708849 EGAZ00001253013, EGAZ00001253091, EGAZ00001253099, EGAZ00001708820, EGAZ00001708842 EGAZ00001253014, EGAZ00001253044, EGAZ00001253094, EGAZ00001708826, EGAZ00001708848 EGAZ00001253052, EGAZ00001253134, EGAZ00001253218, EGAZ00001708821, EGAZ00001708843 EGAZ00001253009, EGAZ00001253015, EGAZ00001253076, EGAZ00001708823, EGAZ00001708845 EGAZ00001253027, EGAZ00001253131, EGAZ00001253160, EGAZ00001708824, EGAZ00001708846 EGAZ00001253132, EGAZ00001253151, EGAZ00001253191, EGAZ00001708815, EGAZ00001708837 EGAZ00001253016, EGAZ00001253168, EGAZ00001253221, EGAZ00001708822, EGAZ00001708844 EGAZ00001253026, EGAZ00001253112, EGAZ00001253159, EGAZ00001708829, EGAZ00001708851 EGAZ00001252877, EGAZ00001252900, EGAZ00001252955, EGAZ00001708817, EGAZ00001708839 EGAZ00001313645, EGAZ00001313749, EGAZ00001313758, EGAZ00001708816, EGAZ00001708838 EGAZ00001313644, EGAZ00001313701, EGAZ00001313764, EGAZ00001708814, EGAZ00001708836 EGAZ00001313829, EGAZ00001313852, EGAZ00001313890, EGAZ00001708818, EGAZ00001708840 EGAZ00001313693, EGAZ00001313759, EGAZ00001313761, EGAZ00001708819, EGAZ00001708841 EGAZ00001313689, EGAZ00001313729, EGAZ00001313751, EGAZ00001708825, EGAZ00001708847 EGAZ00001313963, EGAZ00001314013, EGAZ00001314022, EGAZ00001708831, EGAZ00001708853 EGAZ00001313833, EGAZ00001313903, EGAZ00001313911, EGAZ00001708830, EGAZ00001708852 EGAZ00001314002, EGAZ00001314010, EGAZ00001314027, EGAZ00001708832, EGAZ00001708854 EGAZ00001390301, EGAZ00001390450, EGAZ00001390505, EGAZ00001708834, EGAZ00001708856 EGAZ00001390307, EGAZ00001390443, EGAZ00001390548, EGAZ00001708835, EGAZ00001708857.

## Footnotes

1 GATK Picard http://broadinstitute.github.io/picard.

2
https://github.com/dancooke/starfish.

3
https://github.com/genome/bam-readcount.
