# Supplementary material for: Combinatorial and Machine Learning Approaches for Improved Somatic Variant Calling From Formalin-Fixed Paraffin-Embedded Genome Sequence Data
Source: Front Genet. 2022 Apr 27;13:834764. doi: 10.3389/fgene.2022.834764 (PMC9092826; doi:10.3389/fgene.2022.834764)
Supplement: Supplementary file 2 [file DataSheet1.docx]

### Section 1: Whole-genome sequencing of HTMCP FFPE samples

Submitted FFPE tissue scrolls were transferred into Matrix tubes (Thermo Scientific) for FFPE total nucleic acid (TNA) extraction using the Agencourt FormaPure kit (Beckman Coulter, USA). Briefly, a lysis buffer was added for deparaffinization and lysis. Proteinase K was then added to complete tissue digestion and inactivate nucleases. Samples were then subjected to reverse crosslinking. Binding buffer was added to facilitate immobilization of the nucleic acids to the surface of the paramagnetic beads. Samples were then washed with Ethanol and total nucleic acid eluted in buffer EB.

100 ng of FFPE TNA was arrayed in each well of a 96-well plate and sheared by sonication (Covaris). Sheared DNA was size selected using PCRClean DX magnetic beads (Aline Biosciences) targeting a 200-500 bp fraction. The DNA fragments were repaired, phosphorylated and bead purified in preparation for A-tailing using a custom NEB Paired-End Sample Prep Premix Kit (New England Biolabs). After 3’ A-tailing, Illumina sequencing adapters were ligated for 15 minutes at 20^o^C . Libraries were bead purified and amplified with 8 cycles of PCR using primers containing a hexamer index that enabled library pooling. Library fragment sizes were assessed using an aliquot of PCR amplified library DNA on an Agilent 2100 Bioanalyzer DNA1000 chip, or Caliper GX DNA1000 chip. The final concentration was confirmed by Qubit dsDNA HS Assay. Libraries were sequenced with paired-end 125 bp reads on an Illumina HiSeq2500 instrument.

### Section 2: Commands for each tool

Mutect2:

| #input parameter order: normal bam, output name  #Panel of normals  gatk-package-4.1.4.0-local Mutect2  -R hg38_no_alt.fa  -I ${1}  --germline-resource gnomad.genomes.r2.1.1.sites.liftover_grch38_noalt_sorted_limited.vcf.gz  -O Mutect2/PONBLGSP/${2}.vcf.gz  --native-pair-hmm-threads 46 --independent-mates  gatk-package-4.1.4.0-local.jar CreateSomaticPanelOfNormals  -vcfs Mutect2/PON-BLGSP/sampleList.list  -O /Mutect2/PONBLGSP/BLGSP_pon.vcf.gz  #Mutect2: Step 1: input parameter order: tumor bam, tumor ID  gatk-package-4.1.3.0-local.jar Mutect2  -R hg38_no_alt.fa  -I ${1}  -germline-resource gnomad.genomes.r2.1.1.sites.liftover_grch38_noalt_sorted_limited.vcf.gz -pon /Mutect2/PON-BLGSP/BLGSP_pon.vcf.gz  --f1r2-tar-gz Mutect2/BLGSP/FFPE/${2}f1r2.tar.gz  -O /Mutect2/BLGSP/FFPE/${2}_unfiltered.vcf  normal=`samtools view -H ${1} \| grep ^@RG \| tr '\t' '\n' \| grep ^SM: \| awk -F ":" '{ print $2 }' \| head -n 1`  gatk-package-4.1.4.0-local.jar Mutect2  -R hg38_no_alt.fa  -I ${1} -I ${2} -normal $normal  -germline-resource gnomad.genomes.r2.1.1.sites.liftover_grch38_noalt_sorted_limited.vcf.gz -pon /Mutect2/PON-BLGSP/BLGSP_pon.vcf.gz  --f1r2-tar-gz /Mutect2/BLGSP/FFPE/${3}f1r2.tar.gz -O /Mutect2/BLGSP/FFPE/${3}_unfiltered.vcf  --native-pair-hmm-threads 46 --independent-mates  gatk-package-4.1.4.0-local.jar LearnReadOrientationModel  -I /Mutect2/BLGSP/FFPE/${1}f1r2.tar.gz  #Mutect2: Step 3: GetPileupSummaries: tumor ID, tumor bam  java -Xms40G -jar gatk-package-4.1.4.0-local.jar GetPileupSummaries -I ${2}  -V somatic-hg38%2Fsmall_exac_common_3.hg38.vcf.gz  -L somatic-hg38%2Fsmall_exac_common_3.hg38.vcf.gz  -O /BLGSP/Mutect/${1}Fresh/${1}_getpileupsummaries.table    #Mutect2: Step 4: CalculateContamination: tumor ID  java -Xms40G -jar gatk-package-4.1.4.0-local.jar CalculateContamination  -I BLGSP/Mutect/${1}Fresh/${1}_getpileupsummaries.table  -tumor-segmentation BLGSP/Mutect/${1}Fresh/${1}_segments.table  -O /BLGSP/Mutect/${1}Fresh/${1}_calculatecontamination.table    #Mutect2: Step 5: Filter Calls  ava -Xms40G -jar gatk-package-4.1.4.0-local.jar FilterMutectCalls  -V BLGSP/Mutect/${1}Fresh/${1}_${2}_unfiltered.vcf  --tumor-segmentation /BLGSP/Mutect/${1}Fresh/${1}_segments.table  --contamination-table /BLGSP/Mutect/${1}Fresh/${1}_calculatecontamination.table  --ob-priors /BLGSP/Mutect/${1}Fresh/${1}_read-orientation-model.tar.gz  -O /BLGSP/Mutect/${1}Fresh/${1}_${2}_filtered.vcf  -R hg38_no_alt.fa |
| --- |

LoFreq:

| lofreq somatic -n ${1} -t ${2}  -f hg38_no_alt.fa --threads 40  -o /LoFreq/${3}  -d dbsnp.vcf.gz |
| --- |

SomVarIUS:

| SomVarIUS call_mutations  --bam ${1}  --ref hg38_no_alt.fa  --out /SomVarIUS/${2}.vcf  --dbsnp_bed dbsnp_hg38.bed --ref_filter |
| --- |

Platypus:

| Python2.7 /Platypus_0.8.1/Platypus.py callVariants  --genSNPs=1 --genIndels=0  -o /Platypus/${3}_Fresh.vcf  --refFile=hg38_no_alt.fa  --bamFiles=${1},${2}  --nCPU=35 |
| --- |

Pisces:

| dotnet GeminiMulti.dll  --bam ${1}  --genome /Hg38/  --samtools samtools  --outFolder /Pisces/BLGSP/${2}/  --exepath /Gemini_5.2.10.49/Gemini.dll |
| --- |

Shimmer:

| perl shimmer.pl ${1} ${2}  --ref hg38_no_alt.fa  --outdir /Shimmer/${3} |
| --- |

OutLyzer:

| Python outLyzer.py calling  -bam ${1} -ref hg38_no_alt.fa  -bed ${2}FFPENew.bam.bed -cut 20 -core 10  -samtools samtools  -output /Outlyzer/BLGSP/FFPE |
| --- |

Strelka2:

| configManta.py --normalBam ${1} --tumorBam ${2}  --referenceFasta hg38_no_alt.fa  --runDir /Strelka2/${3}Manta/  python2.7 /Strelka2/${3}Manta/runWorkflow.py  configureStrelkaSomaticWorkflow.py  --normalBam ${1} --tumorBam ${2}  --referenceFasta hg38_no_alt.fa  --indelCandidates /Strelka/${3}Manta/results/variants/candidateSmallIndels.vcf.gz -  -runDir /Strelka/${3}Strelka/  python2.7 /Strelka/${3}Strelka/runWorkflow.py -m local -j 35 |
| --- |

Virmid:

| jar Virmid.jar  -R hg38_no_alt.fa  -N ${1} -D ${2}  -w /Virmid -o ${3} -t 40 |
| --- |

Octopus:

| normal=`samtools view -H ${1} \| grep ^@RG \| tr '\t' '\n' \| grep ^SM: \| awk -F ":" '{ print $2 }' \| head -n 1`  octopus  -R g38_no_alt.fa  -I ${1} ${2} --normal-sample $normal  --threads 47  -o /Octopus/BLGSP/${3}.vcf |
| --- |

### Section 3: Elimination of SomVarIUS and Platypus

When compared against the dbSNP [46] database, Platypus-derived SNVs had an overlap rate of 88.6% and 95% for BLGSP and HTMCP. Platypus was therefore eliminated from further matched normal testing. SomVarIUS variants had near 0 overlap between the paired FF and FFPE results and was eliminated from further tests.

###

### Section 4: Feature importance for FFPolish

###
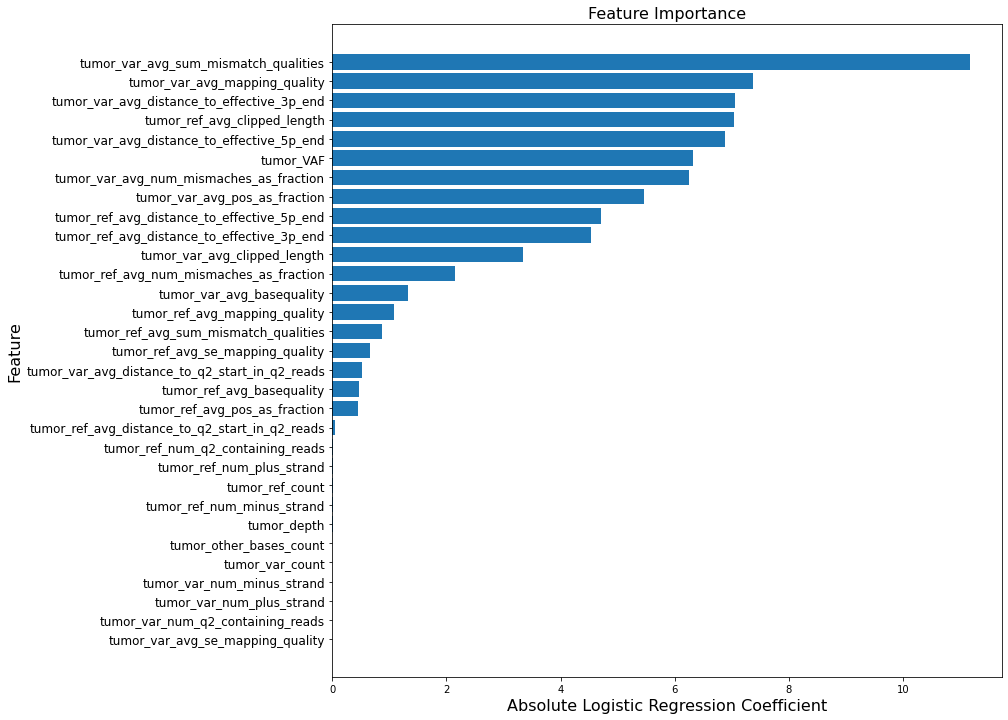


Figure 1: Feature importance of each included feature, calculated by the absolute coefficient from the trained logistic regression model. A higher coefficient corresponds to an important feature. A number of features are of no importance, however, are kept in the model for consistency.


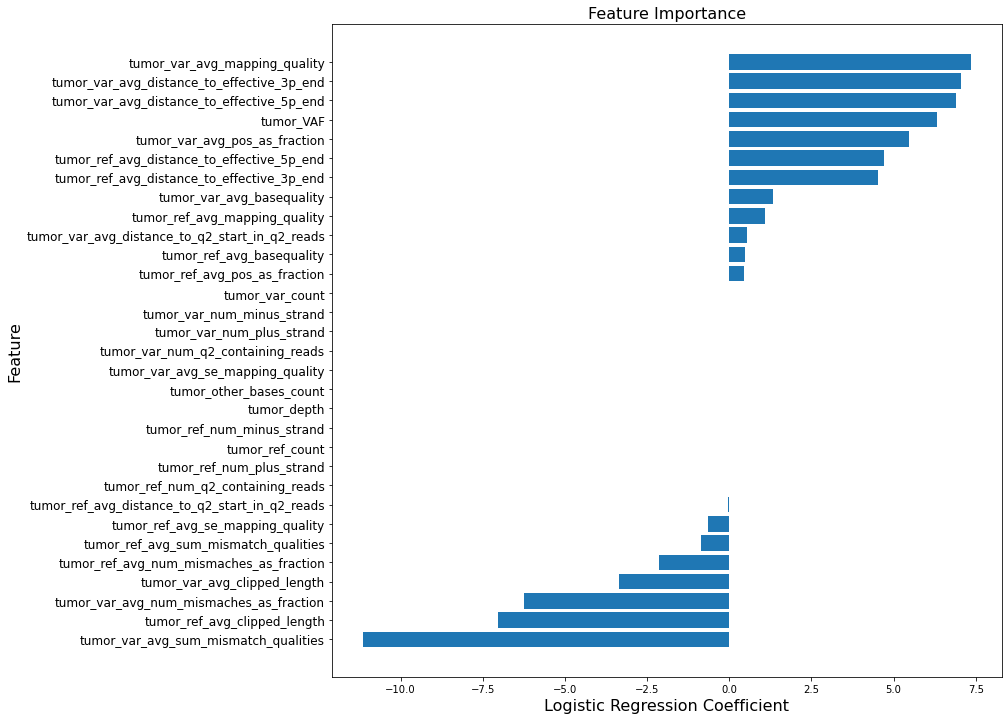


### Figure 2: Feature importance of each included feature, calculated by the coefficient from the trained logistic regression model. This shows the direction of which a feature is important. For example, we see that the coefficient for the average mapping quality for variant reads is positive, therefore a higher mapping quality is indicative that a variant may be legitimate. However, the coefficient for the average sum of base qualities of mismatches (for this feature, mismatches are considered as any allele other than the variant allele) for variant reads is negative, meaning that a high value is indicative that a variant may be an artifact.

### Section 5: Correlations of variant callers and recall^est^ and precision^est^

*
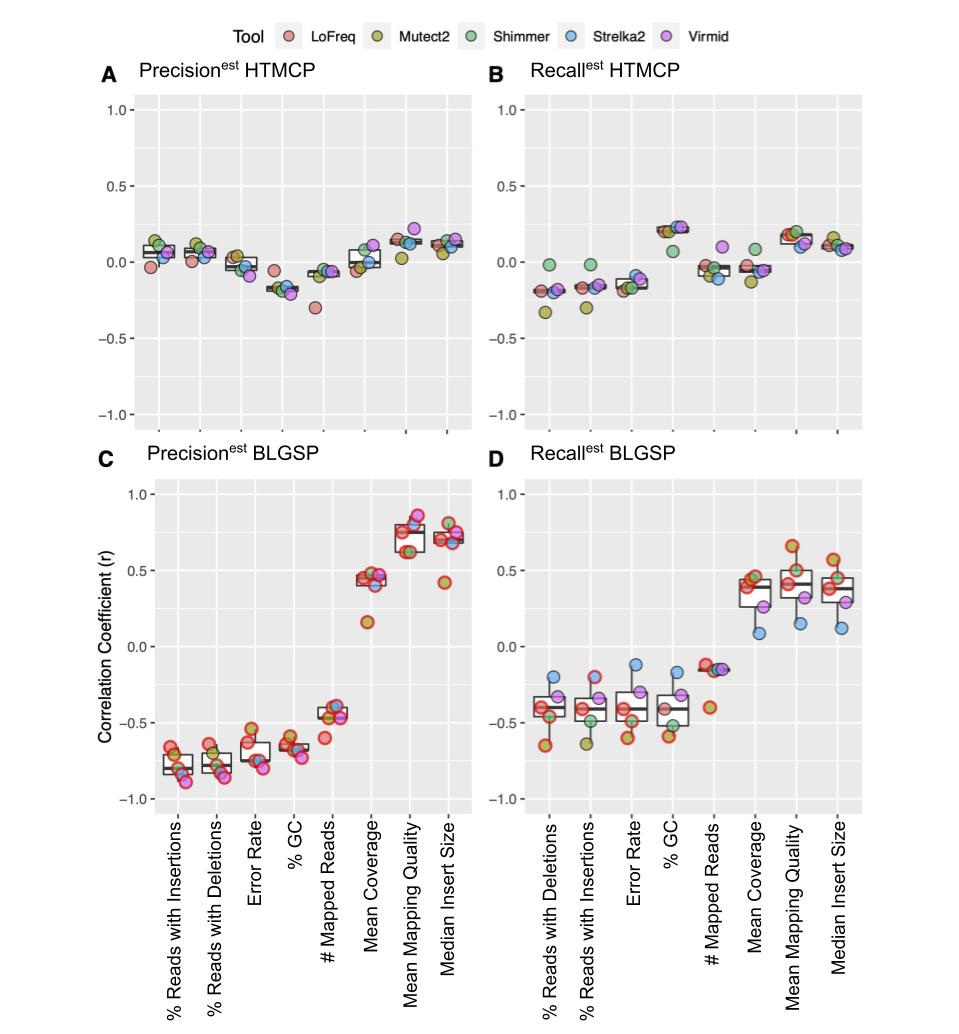
*

***Figure 3:*** *Pearson Correlation of SNV calling (sensitivity and precision) between results of LoFreq, Strelka2, Mutect2, Shimmer and Virmid and quality metrics. Each point is the median value of the metric across all samples. Significant correlates (p≤0.05) are indicated by a red border.*

### Section 6: Benchmarking existing SNV callers on FFPE samples

Tools were tested on FFPE tissue samples using the FF matched normal and then compared to the ground truth data, (FF variants called by Mutect2 and Strelka2). LoFreq and Strelka2 had the highest median precision^est^ and recall^est^, respectively, outperforming other tools.


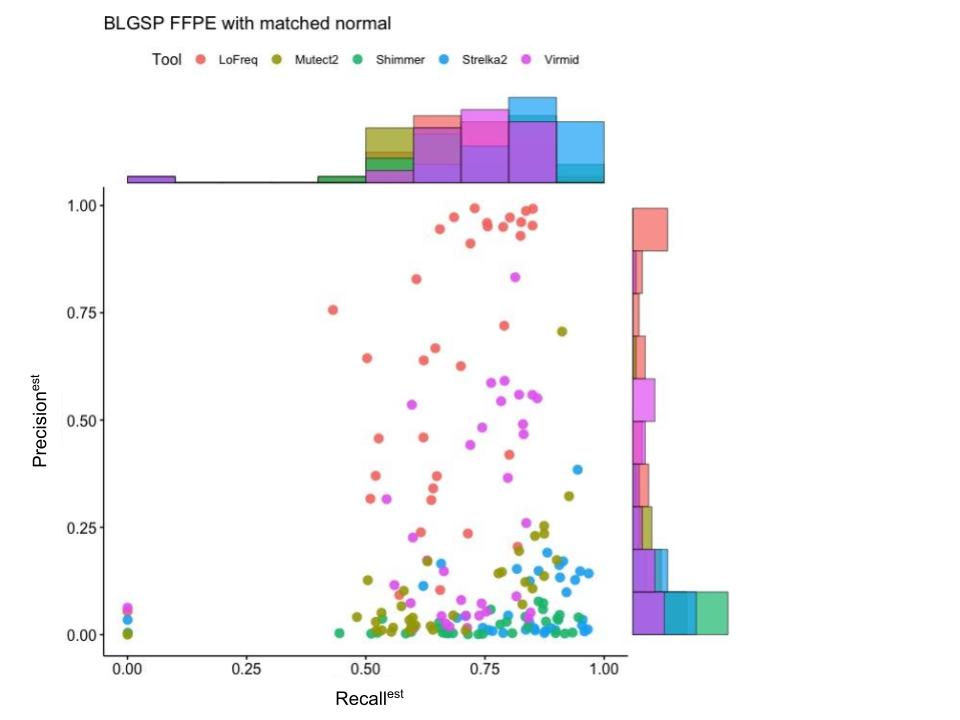


***Figure 4***: *Benchmarking results from BLGSP cohort using LoFreq, Strelka2, Mutect2. Shimmer and Virmid.*

*
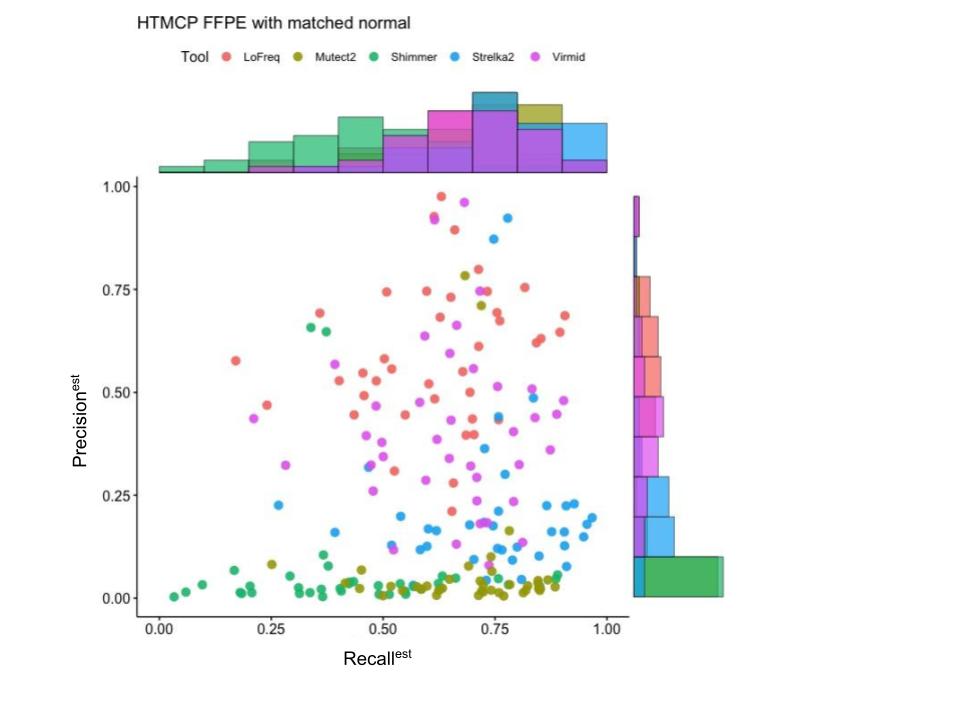
*

***Figure 5:*** *Benchmarking results from HTMCP cohort using LoFreq, Strelka2, Mutect2. Shimmer and Virmid.*

### Section 7: Results in comparison with Brienen et. al.


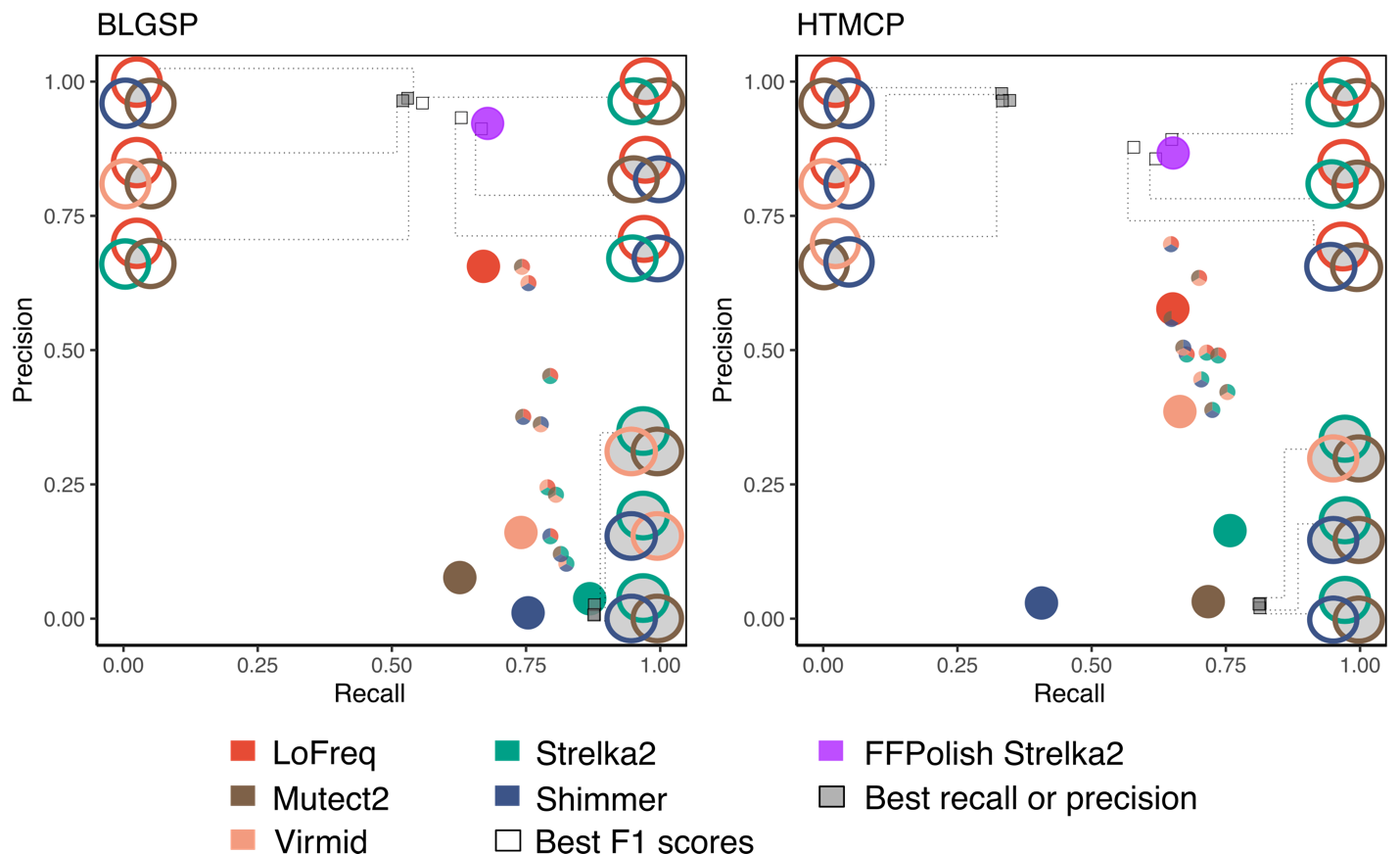


***Figure 6****: Results of tools tested in isolation (solid circles) compared to the combinations that generate the top three values for sensitivity, precision and F1 scores (hollow circles in Venn diagrams). The regions of the venn diagram used are shaded in grey. The pie plots represent the tools used to test the “at least two” strategy from the study of Brienen et al.*

### Section 8: Computational resources for FFPolish and individual tools


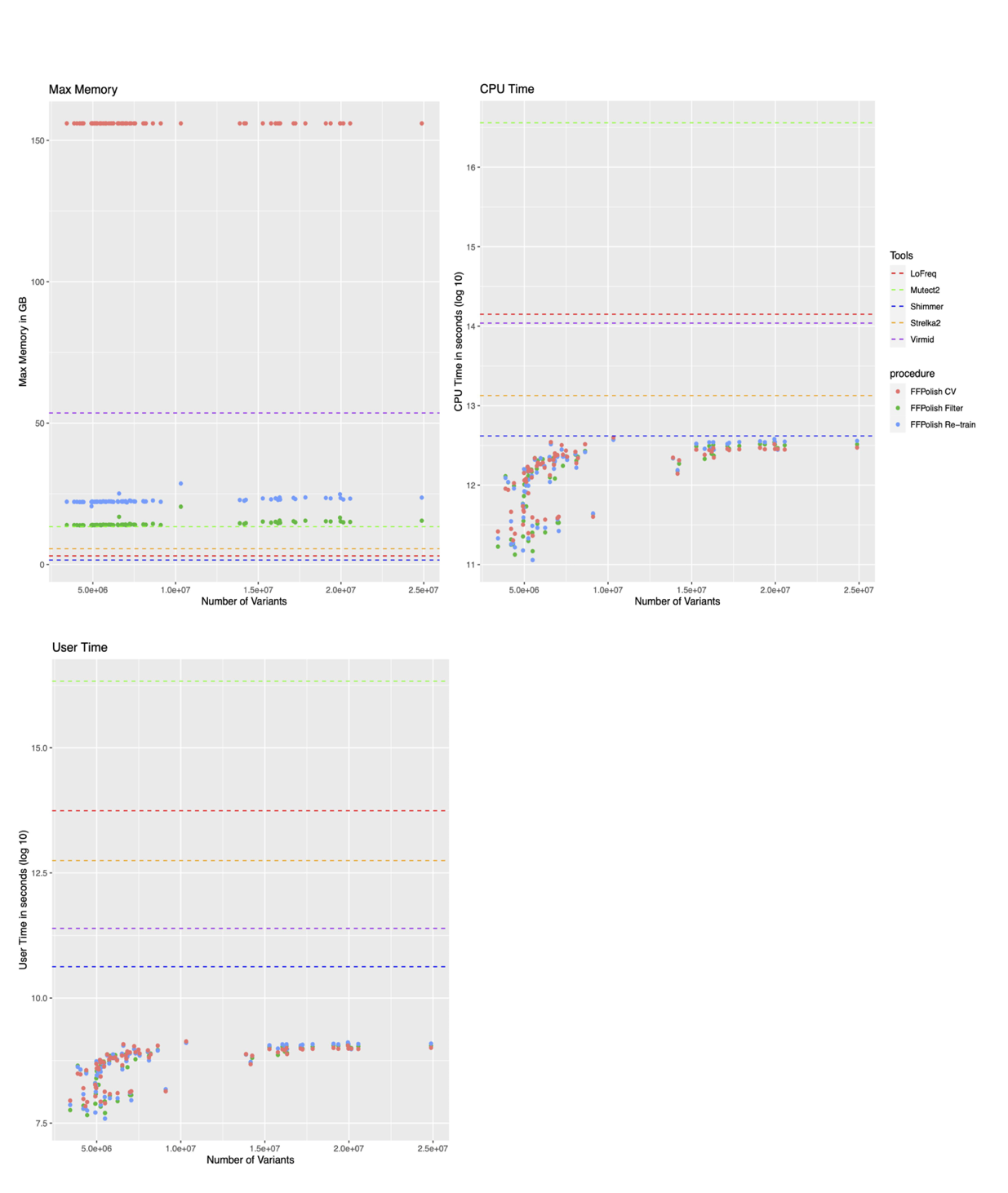


***Figure 7****: The median user time (in seconds), CPU time (in seconds), and maximum memory (in GB) across 9 randomly selected samples required by each of the five tools used in combinations are represented with dashed lines for the respective tool. Computational resources for FFPolish are measured against the number of variants called by Strelka2. ‘FFPolish Filter’ is the default functionality of FFPolish which filters a VCF using the pre-trained model. ‘FFPolish re-train’ refers to retaining the model with added labelled data but using previously optimized hyperparameters. ‘FFPolish CV’ refers to retraining the model while redoing hyperparameter optimization via cross validation.*
